# Supplementary material for: Senescence-associated lineage-aberrant plasticity evokes T-cell-mediated tumor control
Source: Nat Commun. 2025 Mar 31;16:3079. doi: 10.1038/s41467-025-57429-x (PMC11955568; doi:10.1038/s41467-025-57429-x)
Supplement: Supplementary file 1 — Supplementary Information [file 41467_2025_57429_MOESM1_ESM.pdf]

Senescence-associated lineage-aberrant plasticity evokes T-cell-mediated tumor control

Dimitri Belenki[1,2], Paulina Richter-Pechanska[1], Zhiting Shao[1], Animesh Bhattacharya[1], Andrea Lau[1], José Américo Nabuco Leva Ferreira de Freitas[3], Gregor Kandler[1], Timon P. Hick[1,4], Xiurong Cai[1], Eva Scharnagl[5], Aitomi Bittner[1], Martin Schönlein[1,6,7], Julia Kase[1], Katharina Pardon[1], Bernadette Brzezicha[8], Nina Thiessen[9], Oliver Bischof[3], Jan R. Dörr[1,10,11], Maurice Reimann[1], Maja Milanovic[1,12,13], Jing Du[14], Yong Yu[5], Björn Chapuy[12], Soyoung Lee[1,5], Ulf Leser[4], Claus Scheidereit[2], Jana Wolf[2,15], Dorothy N. Y. Fan[1,2,13], and Clemens A. Schmitt[1,2,5,13,16*]

[1]Charité - Universitätsmedizin, corporate member of Freie Universität Berlin, Humboldt-Universität zu Berlin, and Berlin Institute of Health, Department of Hematology, Oncology and Tumor Immunology, and Molekulares Krebsforschungszentrum – MKFZ, Campus Virchow Klinikum, Berlin, Germany

[2]Max Delbrück Center for Molecular Medicine in the Helmholtz Association, Berlin, Germany

[3]IMRB, Mondor Institute for Biomedical Research, INSERM U955 – Université Paris Est Créteil, UPEC, Faculté de Médecine de Créteil, Créteil, France

[4]Knowledge Management in Bioinformatics, Institute for Computer Science, Humboldt-Universität zu Berlin, Berlin, Germany.

[5]Johannes Kepler University, Medical Faculty, Linz, Austria

[6]Department of Oncology, Hematology and Bone Marrow Transplantation with Section of Pneumology, University Medical Center Hamburg-Eppendorf, Hamburg, Germany

[7]Research Institute of Molecular Pathology (IMP), Vienna BioCenter (VBC), Vienna, Austria

[8]Experimental Pharmacology & Oncology Berlin-Buch GmbH, Germany

[9]Core Unit Bioinformatics – CUBI, Berlin Institute of Health. Germany

[10]Charité - Universitätsmedizin, corporate member of Freie Universität Berlin, Humboldt-Universität zu Berlin, and Berlin Institute of Health, Department of Pediatric Oncology and Hematology, Charité-Universitätsmedizin Berlin, Germany

[11]Charité - Universitätsmedizin, corporate member of Freie Universität Berlin, Humboldt-Universität zu Berlin, and Berlin Institute of Health, Experimental and Clinical Research Center (ECRC) of the Max Delbrück Center for Molecular Medicine and Charité-Universitätsmedizin Berlin, Germany

[12]Charité - Universitätsmedizin, corporate member of Freie Universität Berlin, Humboldt-Universität zu Berlin, and Berlin Institute of Health, Department of Hematology, Oncology, and Cancer Immunology, Campus Benjamin Franklin, Berlin, Germany

[13]Deutsches Konsortium für Translationale Krebsforschung (German Cancer Consortium), partner site Berlin, Germany

[14]Medical Research Center and Department of Oncology Binzhou Medical University Hospital, 256600 Binzhou, P.R. China

[15]Department of Mathematics and Computer Science, Free University Berlin, Germany

[16]Kepler University Hospital, Department of Hematology and Oncology, Krankenhausstraße 9, 4020 Linz, Austria

*Corresponding author: Clemens A. Schmitt (ORCID ID 0000-0002-4731-2226)

e-mail: clemens.schmitt@charite.de and clemens.schmitt@kepleruniklinikum.at

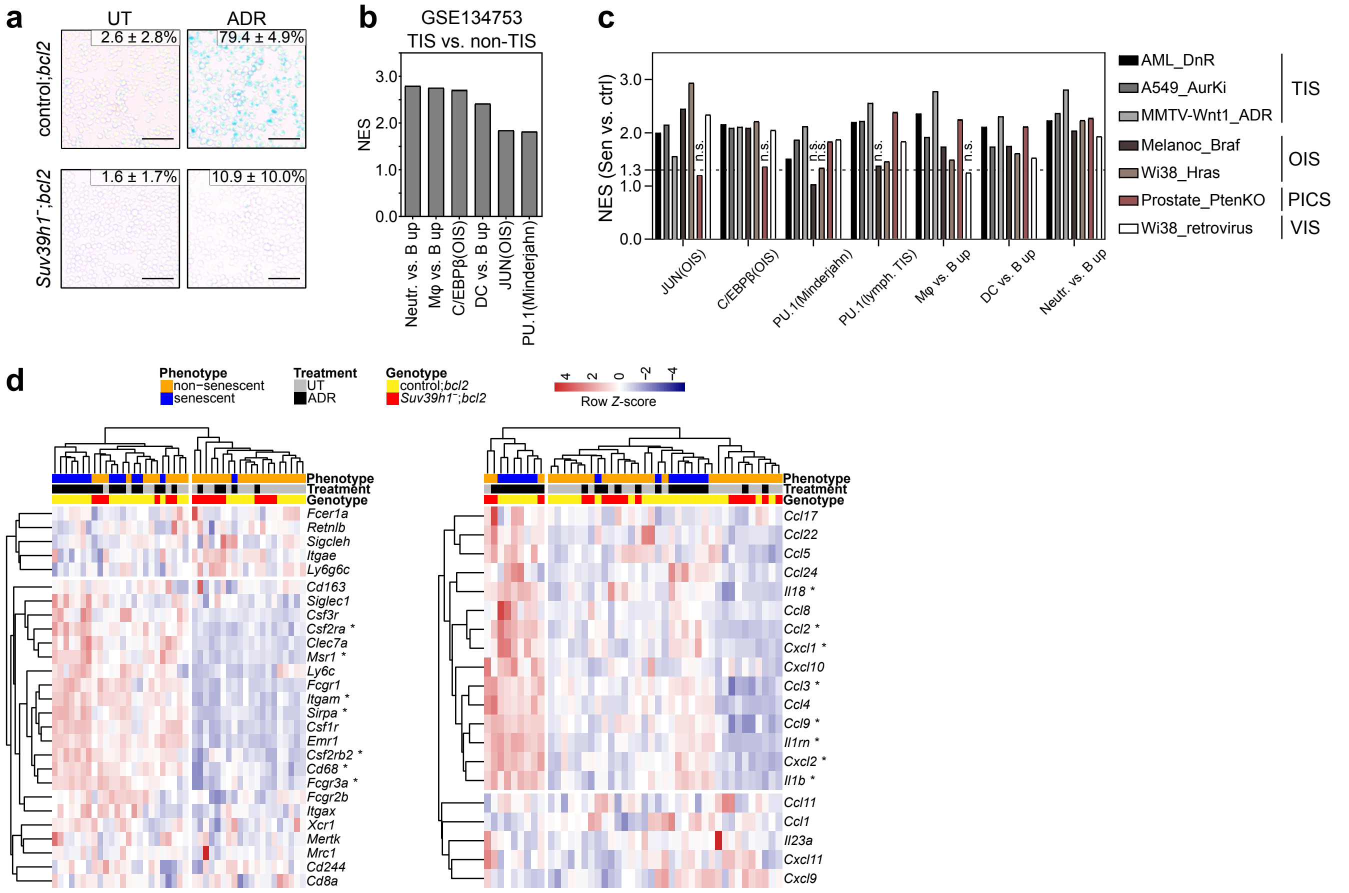

Supplementary Fig. 1: Induction of myeloid lineage-related signatures is a common feature of cellular senescence.

a Senescence-associated β-galactosidase (SA-β-gal) staining of therapy-induced senescence (TIS)-proficient control;*bcl2* and TIS-deficient *Suv39h1*⁻;*bcl2* lymphomas left untreated (UT) or 5-day adriamycin (ADR)-exposed (*n* = 5 independent lymphomas per genotype). Numbers represent mean percentage of positive cells ± SEM. Scale bars, 50 µm. **b** Gene set enrichment analysis (GSEA) of experimentally derived target genes of JUN/AP-1, C/EBPβ and PU.1 transcription factors (TF)[1,2,3], and mouse 'Haemopedia' derived[4] gene sets distinguishing myeloid – i.e. neutrophils, macrophages (Mφ), or dendritic cells (DC) – from B-lymphoid lineages, in ADR-exposed control;*bcl2* (*n* = 14) vs. ADR-exposed *Suv39h1*⁻;*bcl2* (*n* = 8) lymphomas (GSE134753[5]). Positive normalized enrichment scores (NES) indicate enrichment in TIS. False discovery rate (FDR) < 0.05 for all enrichments. **c** GSEA of hematopoietic TF target gene and myeloid signatures (as in **b**) in gene expression profiles (GEP) of various senescence models.

‘PU1(lymph. TIS)’ contains TIS-associated PU.1 targets defined in Supplementary Data 5. Positive NES indicate enrichment in senescent samples. Dashed line indicates threshold of NES = 1.3. FDR < 0.05 for all enrichments except those marked with ‘n.s.’ (not significant). AML_DnR: primary acute myeloid leukemia cells ex vivo 5-day daunorubicin-treated to undergo TIS compared to UT (*n* = 6 individuals each, M.S. and C.A.S., personal communication); A549_AurKi: Aurora kinase inhibitor-induced senescence in human lung cancer A549 cells (GSE102639; *n* = 6 vs. control *n* = 2)[6]; MMTV-Wnt1_ADR: murine MMTV-Wnt1 breast tumors in vivo ADR-treated into TIS or left untreated (GSE133683; *n* = 6 each)[7]; Melanoc_Braf: $Braf^{E600}$ oncogene-induced senescence (OIS) in melanocytes (GSE46801; *n* = 3 vs. control *n* = 6)[8]; Prostate_PtenKO: *Pten* loss induced cellular senescence (PICS) in prostate epithelium vs. wildtype (GSE242433; *n* = 5 each)[9]; Wi38_Hras and Wi38_retrovirus: $Hras^{V12}$-induced (OIS) or retrovirus-induced senescence (VIS) of Wi38 human diploid fibroblasts, respectively (GSE165532; *n* = 3 vs. control *n* = 3, each)[10]. **d** Heatmap and hierarchical clustering using Euclidean distance and complete linkage of commonly used myeloid surface marker (left) and of Mφ-specific cytokine[11,12] (right) genes in the GSE134753 lymphoma TIS data set. * indicates differentially TIS-upregulated genes as in Fig. 1a[5]. Blue tiles in column legend mark senescent samples. Source data provided in Source Data file.

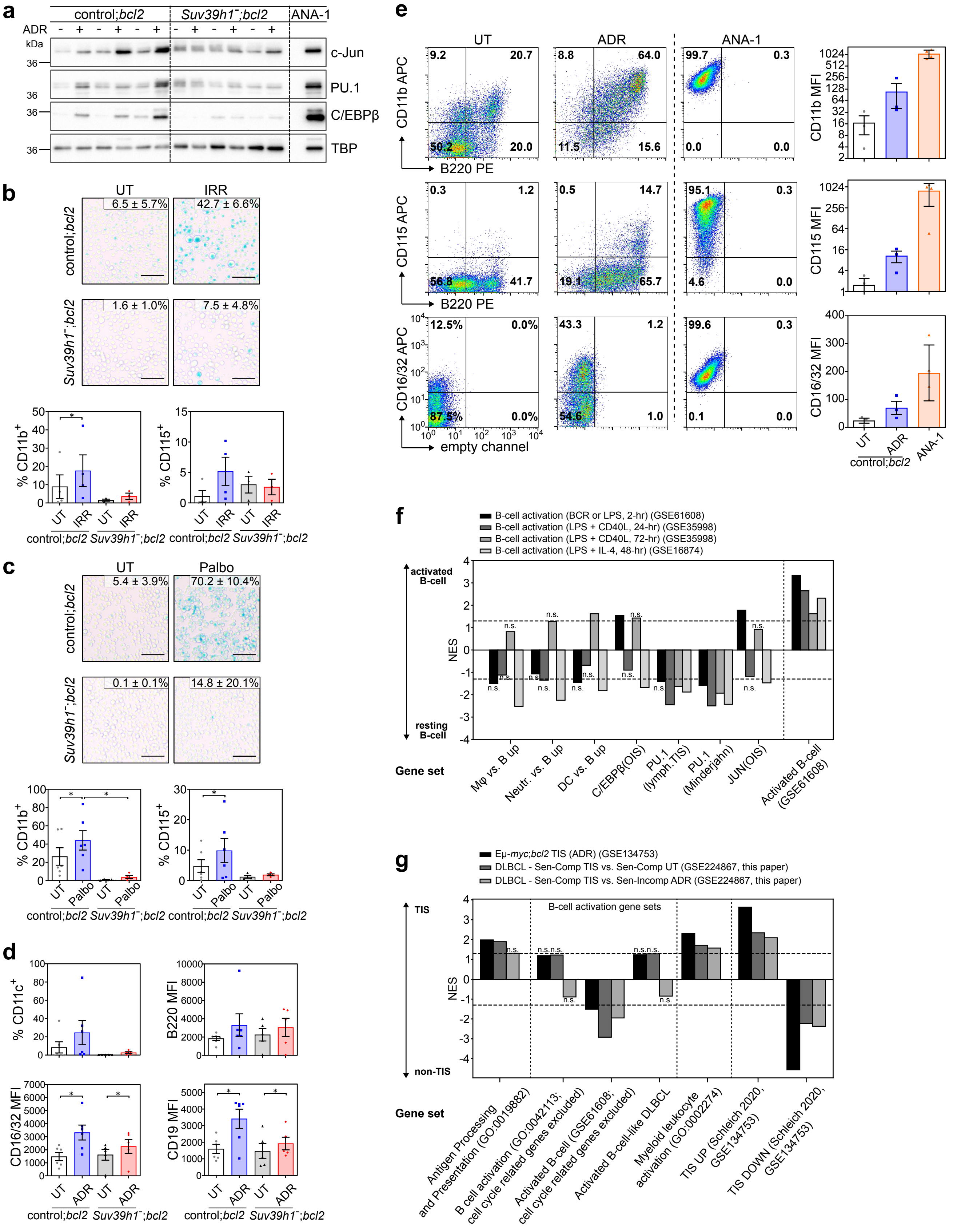
a
control;bcl2
Suv39h1⁻;bcl2
ANA-1
ADR
kDa
36
c-Jun
PU.1
C/EBPβ
TBP
b
UT
IRR
control;bcl2
Suv39h1⁻;bcl2
6.5 ± 5.7%
42.7 ± 6.6%
1.6 ± 1.0%
7.5 ± 4.8%
% CD11b+
% CD115+
UT IRR UT IRR
control;bcl2 Suv39h1⁻;bcl2
c
UT
Palbo
5.4 ± 3.9%
70.2 ± 10.4%
0.1 ± 0.1%
14.8 ± 20.1%
% CD11b+
% CD115+
UT Palbo UT Palbo
d
% CD11c+
B220 MFI
CD16/32 MFI
CD19 MFI
UT ADR UT ADR
e
UT
ADR
ANA-1
CD11b APC
CD115 APC
CD16/32 APC
B220 PE
empty channel
9.2 20.7 50.2 20.0
8.8 64.0 11.5 15.6
99.7 0.3 0.0 0.0
0.3 1.2 56.8 41.7
0.5 14.7 19.1 65.7
95.1 0.3 4.6 0.0
12.5% 0.0% 87.5% 0.0%
43.3 1.2 54.6 1.0
99.6 0.3 0.1 0.0
CD11b MFI
CD115 MFI
CD16/32 MFI
UT ADR ANA-1
control;bcl2
f
B-cell activation (BCR or LPS, 2-hr) (GSE61608)
B-cell activation (LPS + CD40L, 24-hr) (GSE35998)
B-cell activation (LPS + CD40L, 72-hr) (GSE35998)
B-cell activation (LPS + IL-4, 48-hr) (GSE16874)
activated B-cell
resting B-cell
NES
n.s.
Gene set
Mφ vs. B up
Neutr. vs. B up
DC vs. B up
C/EBPβ(OIS)
PU.1 (lymph.TIS)
PU.1 (Minderjahn)
JUN(OIS)
Activated B-cell (GSE61608)
g
Eμ-myc;bcl2 TIS (ADR) (GSE134753)
DLBCL - Sen-Comp TIS vs. Sen-Comp UT (GSE224867, this paper)
DLBCL - Sen-Comp TIS vs. Sen-Incomp ADR (GSE224867, this paper)
TIS
non-TIS
B-cell activation gene sets
NES
n.s.
Gene set
Antigen Processing and Presentation (GO:0019882)
B cell activation (GO:0042113; cell cycle related genes excluded)
Activated B-cell (GSE61608; cell cycle related genes excluded)
Activated B-cell-like DLBCL
Myeloid leukocyte activation (GO:0002274)
TIS UP (Schleich 2020, GSE134753)
TIS DOWN (Schleich 2020, GSE134753)

Supplementary Fig. 2: Induction of myeloid markers is a distinct but common feature of lymphoma cell senescence.

a Immunoblot analyses of nuclear c-Jun, PU.1 and C/EBPβ in untreated (UT) or 5-day adriamycin (ADR)-exposed individual control;*bcl2* and *Suv39h1*$^{-}$;*bcl2* lymphomas (*n* = 3 each). ANA-1 macrophages (Mφ) for comparison. The samples derive from the same experiment but different gels were processed in parallel to probe c-Jun and PU.1, or C/EBPβ. TBP as loading control from the c-Jun/PU.1 blot (see Source Data for TBP from C/EBPβ blot). **b** Senescence-associated β-galactosidase (top) and flow cytometry of CD11b^{+} and CD115^{+} cells (bottom) in individual control;*bcl2* (*n* = 4) and *Suv39h1*$^{-}$;*bcl2* (*n* = 3) lymphomas, either UT or 7 days post-γ-irradiation (IRR). **c** As in **b**, but in control;*bcl2* (*n* = 6) and *Suv39h1*$^{-}$;*bcl2* (*n* = 4) lymphomas, either left UT or 5-day palbociclib-exposed (Palbo). **d** Flow cytometry of CD11c^{+} cells or mean fluorescence intensities (MFI) of CD16/32, B220, and CD19 in UT or ADR-exposed control;*bcl2* (*n* = 6) and *Suv39h1*$^{-}$;*bcl2* (*n* = 5) lymphomas. **e** Flow cytometry of CD11b, CD115, CD16/32, and B220 in individual control;*bcl2* lymphomas (*n* = 3) or ANA-1 (*n* = 4 independent measurements). Representative FACS plots (left), and MFI quantification (right). **b**-**e** Values represent mean percentage of positive cells or MFI ± SEM, except for ANA-1 (**e**) where values are mean MFI ± SD. * two-sided $P < 0.05$ by paired *t*-test for UT vs. treated of same genotype, or by unpaired *t*-test for control vs. *Suv39h1*$^{-}$. Scale bars, 50 µm (**b**, **c**). **f** Gene set enrichment analysis (GSEA) of therapy-induced senescence (TIS)-linked myeloid-related gene signatures in gene expression profiles (GEP) of activated vs. resting B-cells across three data sets. Differentially regulated genes in activated B-cells from GSE61608 as a control. **g** GSEA of TIS in mouse (Eμ-myc;*bcl2*) and human (DLBCL) lymphoma cell data sets, probing three B-cell activation gene sets after exclusion of cell cycle related genes. 'Antigen processing', 'Myeloid leukocyte activation' and TIS genes ('TIS UP/DOWN') used as controls. **f**, **g** n.s. – not significant. Dashed line marks normalized enrichment score (NES) threshold of 1.3. Source data are provided in the Source Data file.

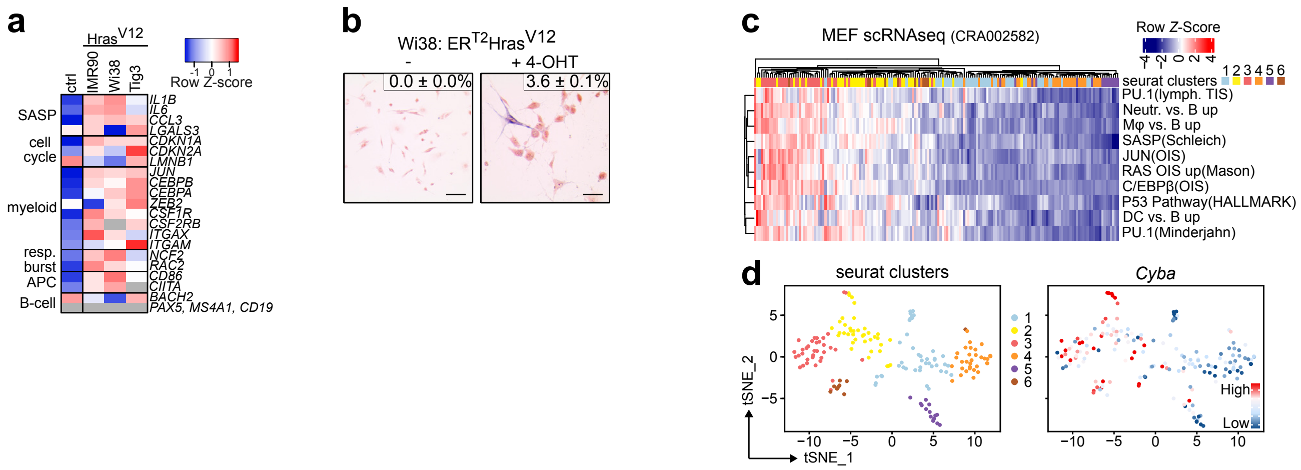

Supplementary Fig. 3: Induction of myeloid features in fibroblast senescence.

a Heatmap of qRT-PCR-determined relative transcript levels in IMR90, Wi38, and Tig3 human diploid fibroblasts undergoing oncogene-induced senescence (OIS) 10 days after $Hras^{V12}$ transduction. *Z*-scores of log_2-transformed fold-changes relative to control (ctrl) transduced samples. Transcripts categorized as indicated; senescence-associated secretory phenotype (SASP), antigen-presenting cell (APC). Grey indicates below-detection levels. **b** Respiratory burst in Wi38 fibroblasts with 4-hydroxytamoxifen (4-OHT)-inducible ER^{T2}-Ras^{V12} after 10-day 4-OHT treatment (OIS) or non-senescent solvent control ($n = 3$ technical replicates). Values represent mean percentage of positive cells ± SD. Scale bars, 100 μm. **c** Heatmap of AUCell enrichments probing gene signatures as in Supplementary Fig. 1c and additional senescence gene sets in scRNA-seq of 175 mouse embryonic fibroblasts (MEF), half of which were senescent[13]. **d** tSNE clustering (left) and projection of transcript levels for respiratory burst gene *Cyba* (bottom right) in MEF as in **c**. Only *Cyba* had sufficient read counts out of five central respiratory burst genes (the other four being *Cybb*, *Ncf1*, *Ncf2*, *Ncf4*). Source data are provided in the Source Data file.

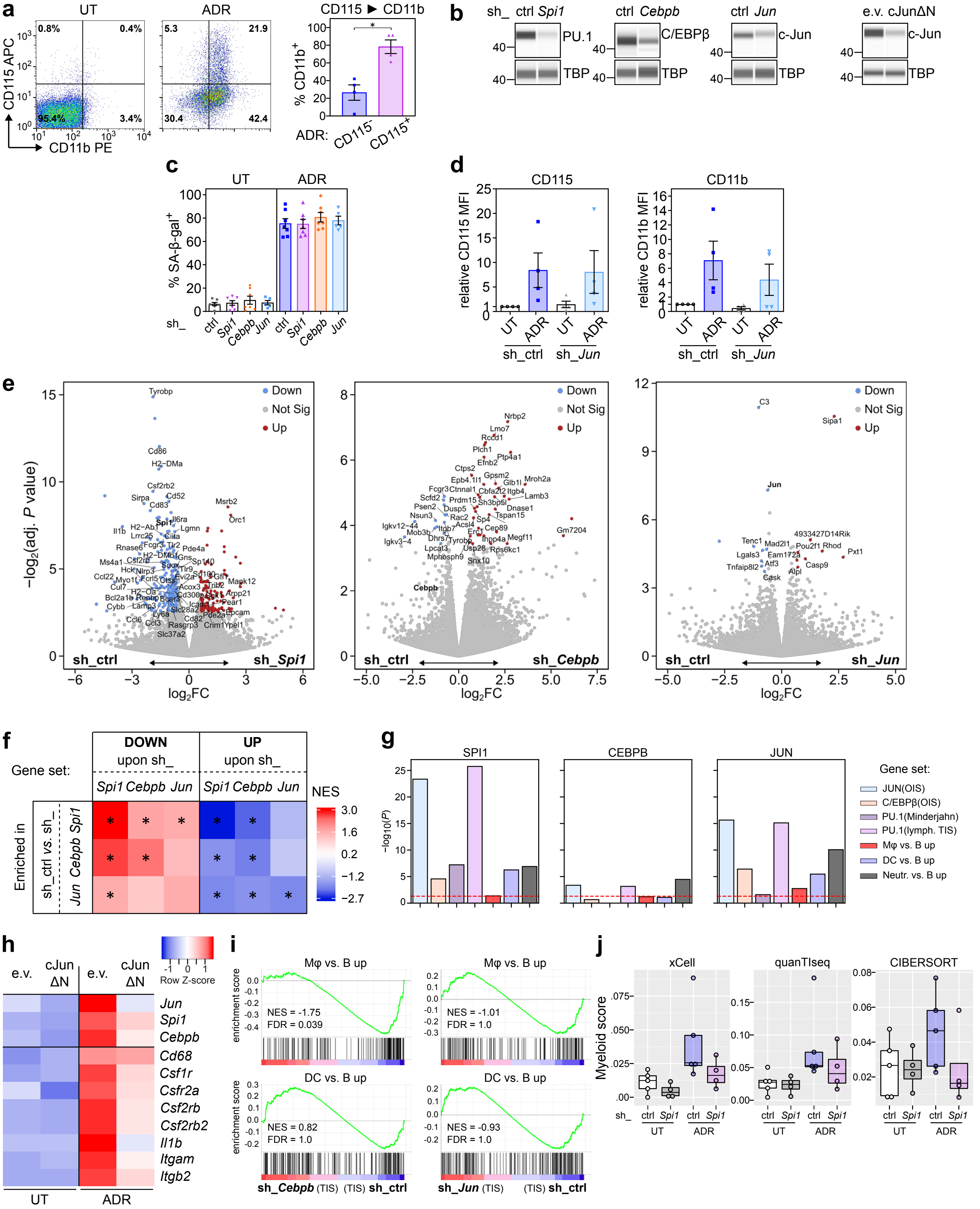
a
UT
ADR
CD115 ► CD11b
CD115 APC
CD11b PE
% CD11b+
ADR: CD115- CD115+
b
sh_ ctrl Spi1
PU.1
TBP
ctrl Cebpb
C/EBPβ
ctrl Jun
c-Jun
e.v. cJunΔN
c
UT
ADR
% SA-β-gal+
sh_
ctrl Spi1 Cebpb Jun
d
CD115
CD11b
relative CD115 MFI
relative CD11b MFI
sh_ctrl
sh_Jun
e
Down
Not Sig
Up
−log2(adj. P value)
sh_ctrl
sh_Spi1
sh_Cebpb
sh_Jun
log2FC
f
DOWN
upon sh_
UP
upon sh_
Gene set:
Spi1 Cebpb Jun
NES
Enriched in
sh_ctrl vs. sh_
g
SPI1
CEBPB
JUN
−log10(P)
Gene set:
JUN(OIS)
C/EBPβ(OIS)
PU.1(Minderjahn)
PU.1(lymph. TIS)
Mφ vs. B up
DC vs. B up
Neutr. vs. B up
h
e.v.
cJun ΔN
Row Z-score
Jun
Spi1
Cebpb
Cd68
Csf1r
Csfr2a
Csf2rb
Csf2rb2
Il1b
Itgam
Itgb2
UT
ADR
i
Mφ vs. B up
DC vs. B up
enrichment score
NES = -1.75
FDR = 0.039
NES = -1.01
FDR = 1.0
NES = 0.82
FDR = 1.0
NES = -0.93
FDR = 1.0
sh_Cebpb (TIS)
(TIS) sh_ctrl
sh_Jun (TIS)
j
xCell
quanTIseq
CIBERSORT
Myeloid score
sh_
ctrl Spi1
UT
ADR

Supplementary Fig. 4: Contributions of distinct master TF to myeloid plasticity in TIS.

a Representative flow cytometry of a 5-day adriamycin (ADR)-exposed or untreated (UT) control;*bcl2* lymphoma, co-stained for CD11b and CD115 (left). Quantification of $CD11b^{+}$ cells after gating on $CD115^{+}$ and $CD115^{-}$ lymphoma subpopulations (right; *n* = 4 independent lymphomas). **b** Capillary immunoassay showing knockdown efficiency of shRNA constructs (representative of *n* = 3) and dominant negative cJunΔN (representative of *n* = 7) in control;*bcl2* lymphomas. Same sh_ctrl used for the three left blots. e.v., empty vector. **c** SA-β-gal in control;*bcl2* lymphomas with shRNAs targeting *Spi1* (*n* = 7), *Cebpb* (*n* = 7), *Jun* (*n* = 5)*,* or non-targeting (ctrl; *n* = 7), after 5-day ADR-exposure or UT. **d** Flow cytometry of CD11b and CD115 in UT and 5-day ADR-exposed control;*bcl2* lymphomas with sh_ctrl or sh_*Jun* (*n* = 4 each). Mean fluorescence intensity (MFI) relative to UT sh_ctrl. **a**, **c**, **d** Bars show mean percentage or mean fold expression ± SEM. * $P < 0.05$ by two-sided, paired *t*-test. **e** Significantly downregulated (blue) and upregulated (red) genes in RNA-seq of ADR-treated therapy-induced senescent (TIS) control;*bcl2* lymphomas as in **c** (*n* = 4 each; Supplementary Data 3). **f** Heatmap of gene set enrichment analyses (GSEA) comparing RNA-seq data from **e**, marking overlapping control of target gene sets across transcription factors (TF). Gene sets affected by specific TF depletion in columns (cf. Supplementary Data 3). Positive normalized enrichment scores (NES) (red) indicate enrichment in TIS sh_ctrl, negative NES (blue) in TIS sh_TF. * false discovery rate (FDR) < 0.05. **g** Hypergeometric *P* values for JASPAR-identified TF binding sites gaining ATAC-seq signal in TIS (*n* = 2, cf. Fig. 1d) for myeloid-related signatures (cf. Supplementary Fig. 1b). Red dashed line marks threshold of $P = 0.05$. **h** qRT-PCR expression analysis of TF (top) and myeloid-related genes (bottom) in control;*bcl2* lymphomas with cJunΔN or e.v. (sample numbers in Source Data). **i** GSEA of Mφ and DC signatures comparing *Cebpb* (left) or *Jun* (right) depletion to sh_ctrl in TIS (as in **e**; *n* = 4 each). Negative NES indicate enrichment in sh_ctrl TIS. **j** Immune deconvolution of RNA-seq data comparing TIS vs. UT control;*bcl2* lymphomas, showing decreased myeloid pseudo-presence in PU.1-depleted (sh_*Spi1; n* = 4) vs. sh_ctrl (*n* = 5) specimens. Boxplots show

total enrichment from xCell or total cell fractions from quanTIseq and CIBERSORT. Source data in Source Data file.

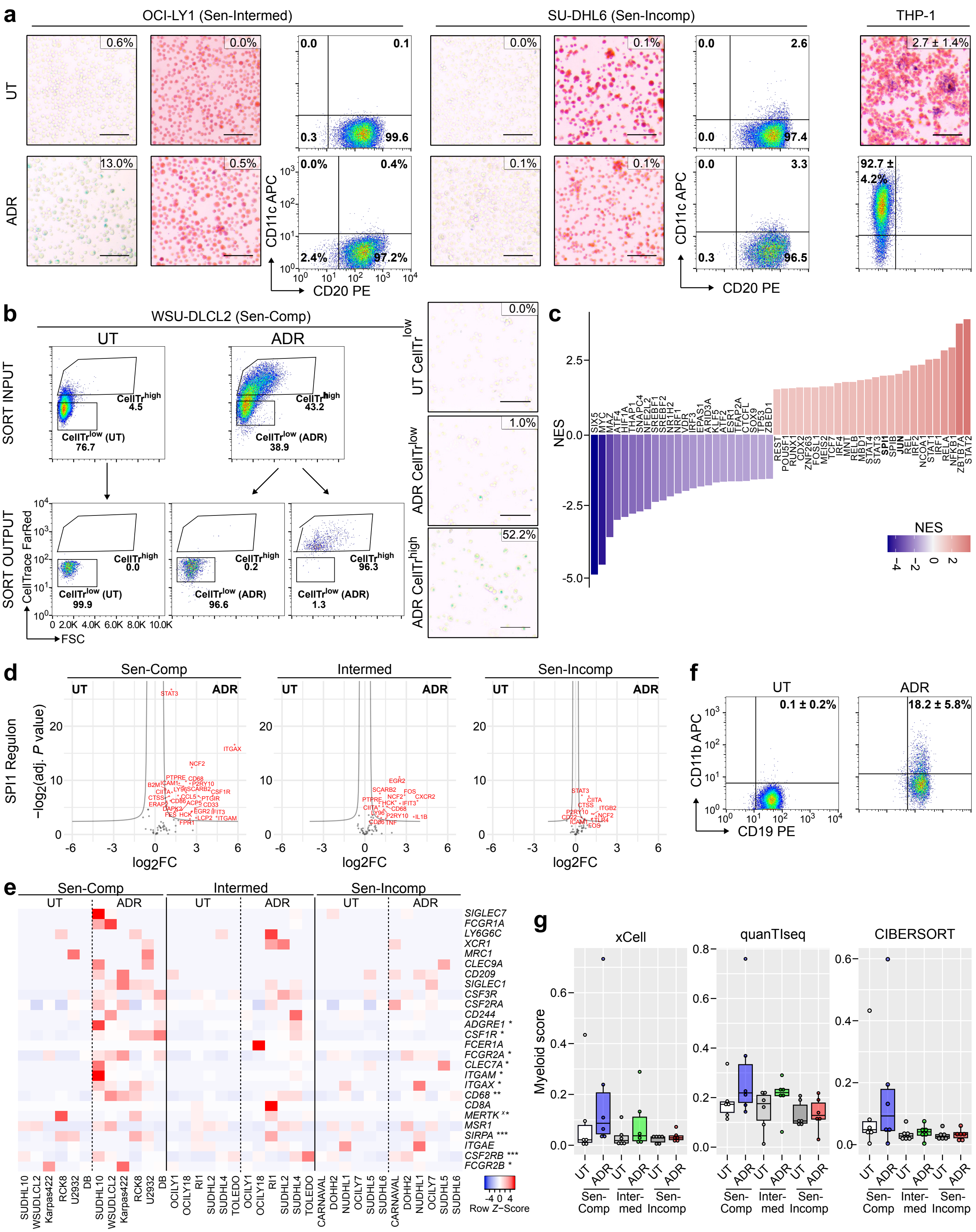
a
OCI-LY1 (Sen-Intermed)
SU-DHL6 (Sen-Incomp)
THP-1
UT
ADR
0.6%
0.0%
13.0%
0.5%
0.0%
0.1%
0.1%
0.1%
2.7 ± 1.4%
92.7 ± 4.2%
CD11c APC
CD20 PE
b
WSU-DLCL2 (Sen-Comp)
SORT INPUT
SORT OUTPUT
CellTrace FarRed
FSC
UT CellTr low
ADR CellTr low
ADR CellTr high
c
NES
d
Sen-Comp
Intermed
Sen-Incomp
SPI1 Regulon
−log2(adj. P value)
log2FC
e
Row Z-Score
f
CD11b APC
CD19 PE
0.1 ± 0.2%
18.2 ± 5.8%
g
xCell
quanTIseq
CIBERSORT
Myeloid score
Sen-Comp
Inter-med
Sen-Incomp

Supplementary Fig. 5: TIS-associated myeloid differentiation features are recapitulated in DLBCL cell lines.

a Senescence-associated β-galactosidase (SA-β-gal) staining, respiratory burst, and CD11c/CD20 flow cytometry of 5-day adriamycin (ADR)-exposed vs. drug-naïve (UT) OCI-LY1 (senescence-intermediate) and SU-DHL6 (senescence-incompetent) diffuse large B-cell lymphoma (DLBCL) cells. THP-1 as control (respiratory burst and CD11c). Values in microscopy images represent the percentage of marker-positive cells as individually plotted in Fig. 4a-b. Mean $CD11c^{+}$ percentage ± SD in flow plot for THP-1 (*n* = 3 independent measurements). **b** FACS sorting of ADR-exposed senescence-enriched ($CellTrace^{high}$ vs. $CellTrace^{low}$) and UT senescence-depleted ($CellTrace^{low}$) DLBCL cells. Gating strategy, purity assessment (left; percentages of gated populations shown) and SA-β-gal positivity for sorted senescence-competent WSU-DLCL2 cells (right). Data for all cell lines in Supplementary Data 4. **a**, **b** Scale bars, 100 μm. **c** DoRothEA transcription factor (TF) footprinting of RNA-seq of ADR $CellTrace^{high}$ and UT $CellTrace^{low}$ DLBCL samples from **b**, comparing senescence-competent to -intermediate groups (*n* = 6 cell lines each). Normalized enrichment scores (NES) for top-50 TF reflecting ADR-induced relative gain (red) or loss (blue) in TF activity in senescence-competent compared to senescence-intermediate cell lines. **d** DoRothEA SPI1 regulon in RNA-seq data from **b**, **c** comparing ADR $CellTrace^{high}$ (ADR) vs. UT $CellTrace^{low}$ (UT) in three senescence-capacity DLBCL cell line groups (*n* = 6 each); red indicates adj. $P < 0.10$. **e** Heatmap of RNA-seq for myeloid surface marker genes in ADR and UT DLBCL cell lines, as in **b**-**d**. Expression correlates with senescence status. * adj. $P < 0.10$ by DESeq2 for paired ADR vs. UT comparisons in senescence-competent cell lines; ** same, but for senescence-competent and -intermediate (or ^{X}* for only the latter); *** same, but for all three groups. **f** Flow cytometry of SU-DHL10 cells (UT or 5-day ADR-exposed) co-stained for CD11b and CD19. Values show mean $CD11b^{+}CD19^{+}$ percentage ± SD (*n* = 3 independent experiments). **g** Myeloid scores from immune deconvolution algorithms of RNA-seq data for DLBCL cell lines as in **b**-**e** (*n* = 6 per category). Boxplots show aggregated myeloid enrichment (xCell) or

total myeloid fractions (quanTIseq, CIBERSORT). **a**, **f** Flow cytometry gates set using isotype controls acquired for UT and ADR samples individually. Source data in the Source Data file.

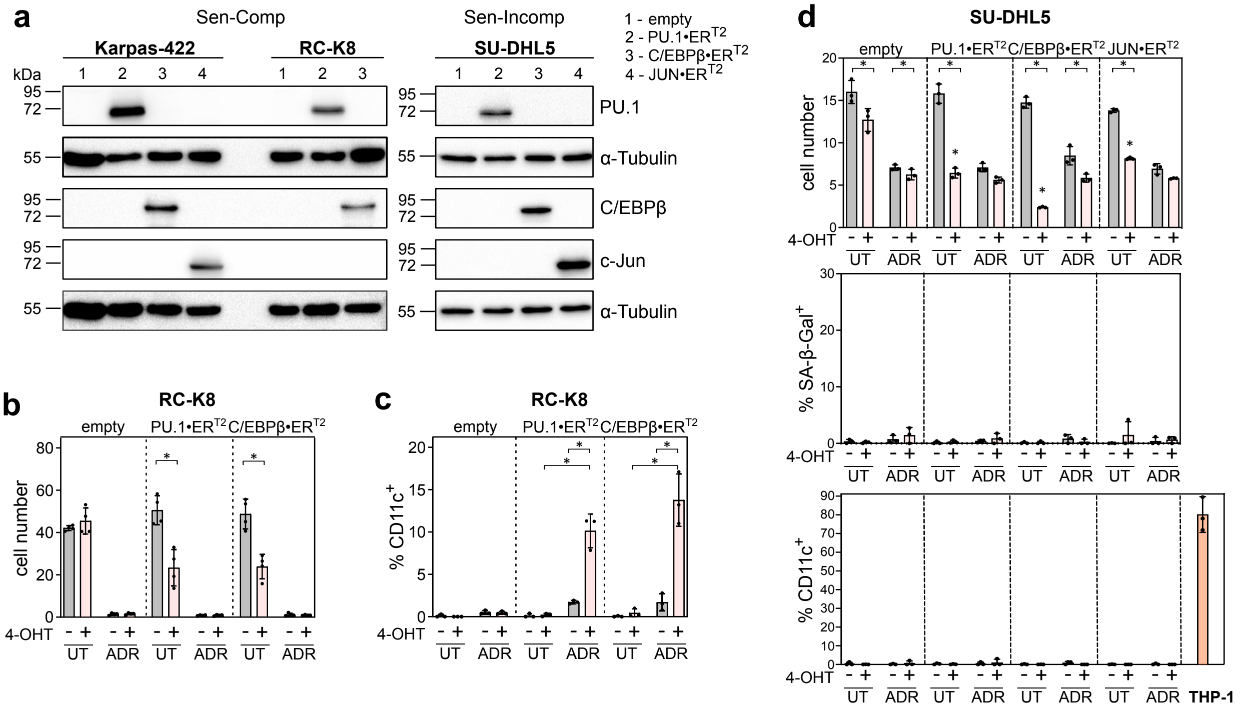

Supplementary Fig. 6: PU.1, C/EBPβ, and AP-1 TF regulate an aberrant myeloid lineage program in DLBCL.

a Immunoblot confirming the expression of PU.1-, C/EBPβ- and JUN-ER^{T2} fusion proteins (predicted molecular weights of 66.6, 72.2 and 71.2 kDa) in senescence-competent (Karpas-422, RC-K8) and -incompetent (SU-DHL5) DLBCL cell lines. α-Tubulin as loading control. The samples derive from the same experiment but different gels were processed in parallel to probe PU.1 (top 2 slices) or C/EBPβ and c-Jun (bottom 3 slices). **b** Proliferation of RC-K8 cells as in **a**, untreated or adriamycin (ADR)-treated (15 ng ml^{-1}), then exposed to 4-hydroxytamoxifen (4-OHT) or solvent (EtOH) at day 3; analyzed 7 days post-ADR. Fold-change in cell number comparing start to end of treatment. Individual dots and mean fold-changes ± SD from *n* = 4 independent experiments. **c** Flow cytometry of samples as in **b**, showing mean percentage of CD11c$^+$ cells ± SD (*n* = 3 independent experiments). **d** Proliferation (top), SA-β-gal percentage (middle), and CD11c (bottom, THP-1 as control) in SU-DHL5 cells as in **a**, treated with solvent, 4-OHT, ADR (3.75 ng ml^{-1}) or ADR + 4-OHT, analyzed 5 days post-treatment. **b-d** * Two-sided, paired

t-test $P < 0.05$. Isolated asterisks denote $P < 0.05$ comparing TF-ER^{T2} + 4-OHT vs. empty vector + 4-OHT (**d**). Source data are provided in the Source Data file.

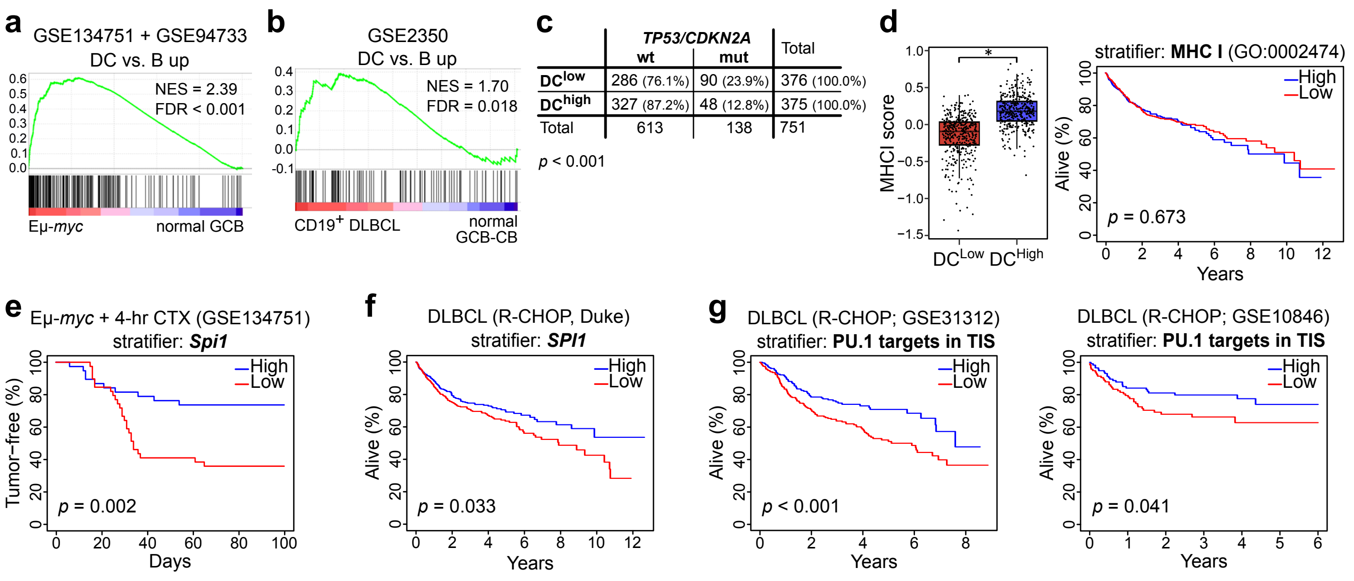

Supplementary Fig. 7: TIS-associated myeloid differentiation features are linked to favorable outcome in DLBCL.

a Gene set enrichment analysis (GSEA) using the mouse ‘DC vs. B-cell up‘ gene set probing gene expression profiles (GEP) of whole lymph node lysates of Eμ-*myc* lymphomas (GSE134751[5], *n* = 20) and reactive germinal center (GC) B-cells purified from wild-type C57BL/6 mice (GSE94733; *n* = 12). **b** GSEA of the human ‘DC vs. B-cell up‘ gene set probing $CD19^+$ cells from primary diffuse large B-cell lymphoma (DLBCL) samples (*n* = 7) vs. normal tonsil GC B-cells ($CD77^+$ centroblasts; GSE2350; *n* = 10). **a**, **b** Enrichment plots include normalized enrichment score (NES) and false discovery rate (FDR) values. **c** Contingency table of DLBCL cases (Duke cohort), divided into ‘DC vs. B up‘ (DC) signature-high and -low tumors, as in Fig. 5g, and further categorized by mutations and copy number alterations in senescence gatekeeper gene loci *TP53* and *CDKN2A*[14,15]. Significant association between DC^{low} status and *TP53*/*CDKN2A* lesions (mut) indicated by Pearson's χ^2 test *P* value. **d** Average expression scores of “antigen processing and presentation of peptide antigen via MHC class I” gene set (GO:0002474) in DLBCL cases as in **c** (left). * $P < 0.05$ by two-sided Wilcoxon rank-sum test. Kaplan-Meier plot of overall survival (OS) for the same Duke DLBCL patients based on GO:0002472 gene set expression (right). ‘High’ is above median and ‘Low’ is below. **e** Kaplan-Meier plot of tumor-free survival in Eμ-*myc* lymphoma-

bearing mice after a single-dose of cyclophosphamide (CTX), stratified by median *Spi1* expression in 4-hr CTX-challenged lymph nodes (GSE134751; *n* = 77). **f** OS of R-CHOP-treated DLBCL patients (Duke cohort), stratified by *SPI1* expression in pre-therapy biopsies. **g** OS of R-CHOP treated DLBCL patient cohorts from GSE31312 (left, *n* = 470) and GSE10846[16] (right, *n* = 233), stratified by the average expression of the humanized PU.1(TIS) signature (cf. Supplementary Data 5). **d-g** All survival curves reflect a median split with log-rank-test *P* shown. Source data are provided in the Source Data file.

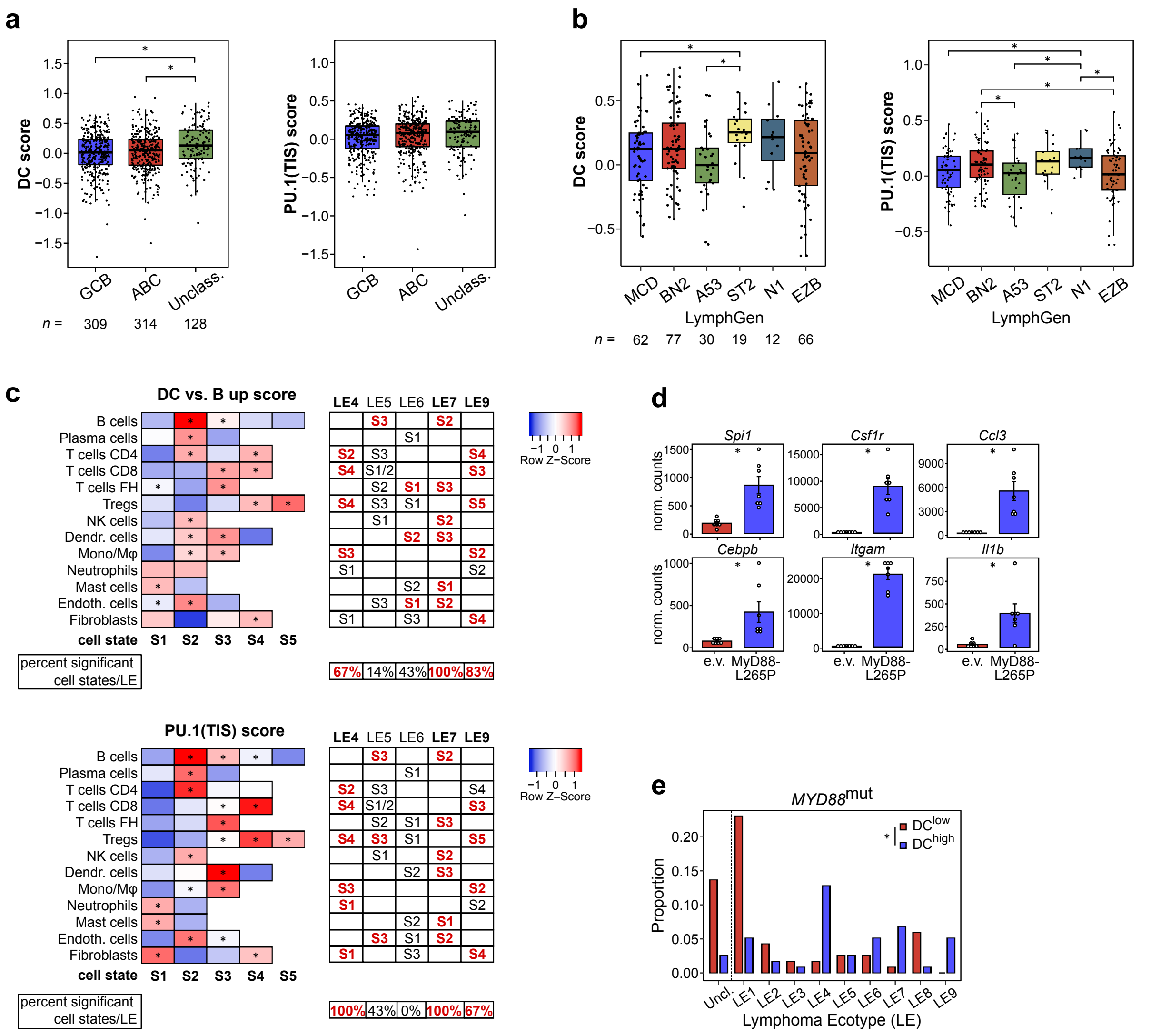

Supplementary Fig. 8: TIS-associated PU.1 and DC signatures correlate with distinct DLBCL ecotypes.

a Boxplots showing distribution of human 'dendritic cell (DC) vs B up' (left) (DC) and humanized 'PU.1 (TIS)' (right) gene signature in the Duke diffuse large B-cell lymphoma (DLBCL) cohort[14], stratified by cell-of-origin status: activated B-cell (ABC), germinal center B-cell (GCB) or unclassified. * Benjamini-Hochberg adj. $P < 0.05$. **b** As in **a**, showing DC and 'PU.1(TIS)' expression in the Schmitz DLBCL cohort, stratified by LymphGen genetic classifier (only unequivocally assigned classes considered)[17,18]. * Benjamini-Hochberg adj. $P < 0.10$. **a**, **b** Sample sizes (*n*) indicated in the figure. Two-sided pairwise Wilcoxon rank-sum test was used. **c** Heatmap of median DC (top) and PU.1(TIS) (bottom) gene expression

scores in Duke DLBCL cohort subgroups, stratified by B-cell and tumor microenvironment cell states (Bcs and TMEcs)[19] as indicated on the left. * in heatmap cells marks significantly higher expression level (Benjamini-Hochberg adj. $P < 0.05$ by pairwise Wilcoxon rank-sum test) in patients assigned to the corresponding cell state (as indicated below by S1 – S5) than at least half of the other patient-assigned cell states for the same cell type. Accompanying tables show reported Bcs/TMEcs composition of selected lymphoma ecotypes (LE) significantly scoring for DC and PU.1(TIS) signatures in Fig. 6a (right column). Cell states scoring significantly as shown in the heatmaps are marked in bold and red. Only LE4, LE7 and LE9 show significantly higher expression of DC and PU.1(TIS) signatures in more than half of the Bcs/TMEcs defining the respective LE (percentages shown below). Note that the DC- and PU.1(TIS)-signature-associated DC and monocytic states are also constituents of LE4, LE7 or LE9. **d** RNA-seq expression levels of PU.1- and C/EBPβ-related genes in individual lymphomas formed in recipient mice transplanted with Eμ-*myc* hematopoietic stem cells with or without stable introduction of a MyD88-L265P lesion (GSE141454[6]; $n = 7$ each). Bar plots show means of normalized counts ± SEM. * adjusted $P < 0.05$ by DESeq2. **e** Frequency of lymphoma ecotypes among MyD88 mutation-positive DLBCL (Duke cohort), divided into DC^{low} ($n = 66$) and DC^{high} ($n = 51$). * Pearson's χ^2 $P < 0.05$. Source data are provided in the Source Data file.

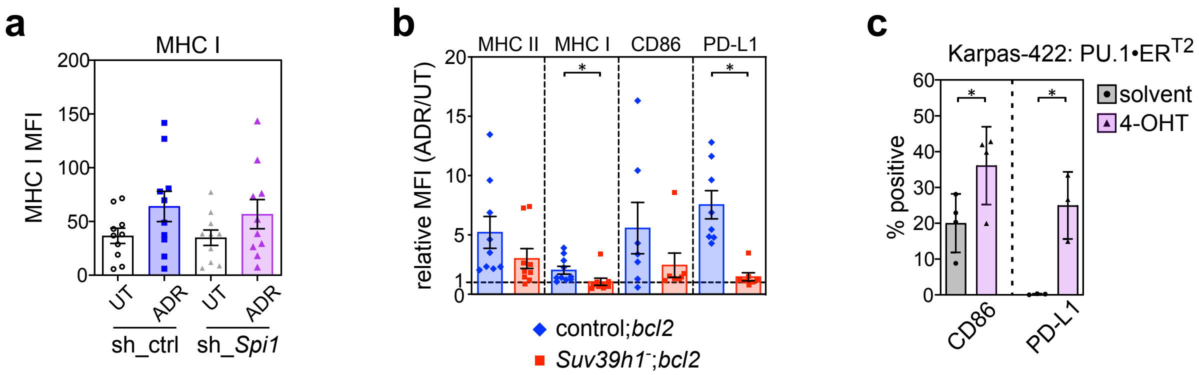

Supplementary Fig. 9: TIS promotes antigen presentation and expression of immune checkpoint molecules.

a MHC class I expression by flow cytometry in individual untreated (UT) and 5-day adriamycin (ADR)-exposed control;*bcl2* lymphomas, transduced with *Spi1* targeting or control shRNA (*n* = 10 each). Data points represent isotype-corrected mean fluorescence intensity (MFI) values; bars show mean values ± SEM. **b** Flow cytometry of MHC class II, MHC class I, CD86 and PD-L1 (CD274) in UT or 5-day ADR exposed individual control;*bcl2* and *Suv39h1*⁻;bcl2 lymphomas. Sample sizes: MCH class II (*n* = 9 each), MHC class I: (*n* = 10 and *n* = 9), CD86 (*n* = 7 each), CD274 (*n* = 8 and *n* = 7) for control;*bcl2* and *Suv39h1*⁻;bcl2 lymphomas, respectively. Data points show relative MFI (ADR/UT); bars show mean values ± SEM. **c** Flow cytometry of CD86 (*n* = 4 independent experiments) and PD-L1 (*n* = 3 independent experiments) on Karpas-422 DLBCL cells transduced with PU.1-ER^{T2} and treated with 4-hydroxytamoxifen (4-OHT) or solvent for 5 days. Bars represent the mean percentage of positive cells ± SD. * $P < 0.05$ by two-sided, unpaired (**b**) or paired (**c**) *t*-test. Source data are provided in the Source Data file.

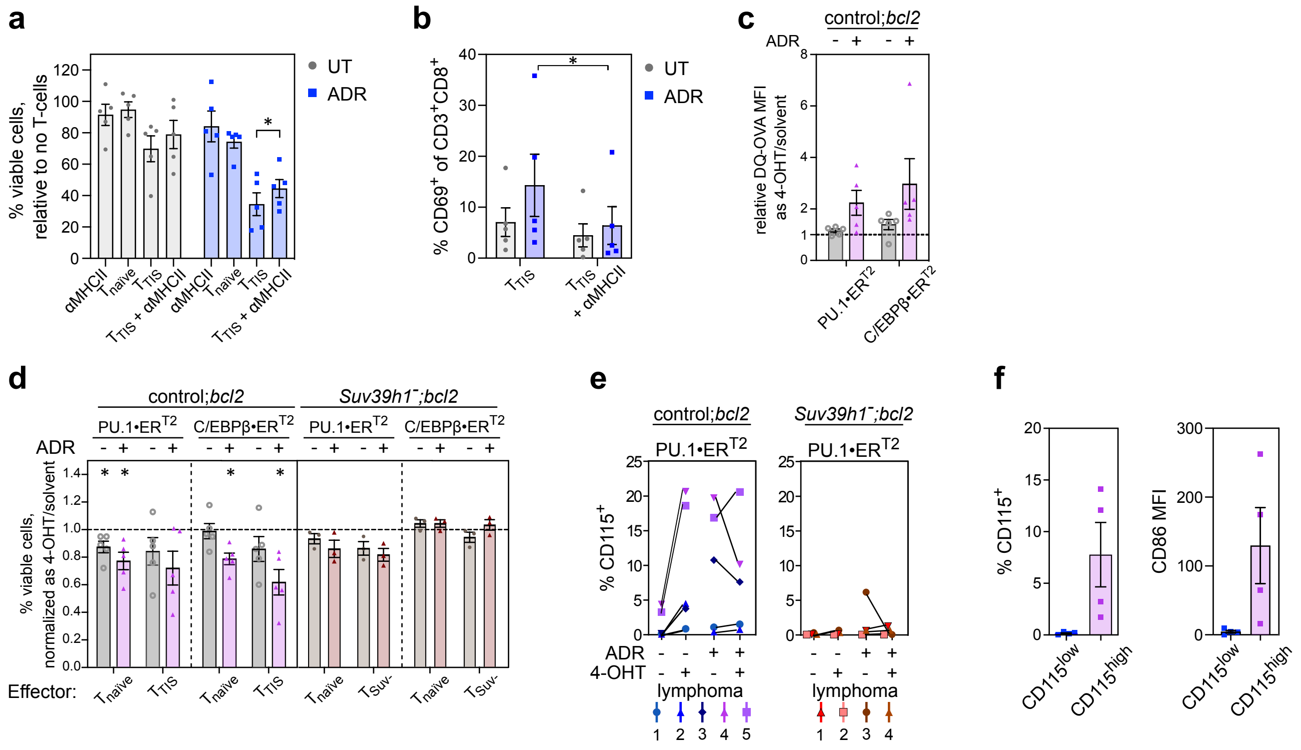

Supplementary Fig. 10: TIS-associated PU.1-evoked T-cell recognition is linked to features of antigen-presenting cells.

a Viability of individual control;*bcl2* lymphomas (*n* = 5) treated with adriamycin (ADR) for 6 days or left untreated (UT), then co-cultured for 48-hr with T_{TIS} alone or in conjunction with an MHC class II-blocking antibody (αMHCII; 5 μg ml^{-1}). $T_{naïve}$ used for comparison (cf. Fig. 8b). Note that MHC class II ligation alone (αMHCII) exerted variable cytotoxicity[20]. Bars represent mean percentage of viable cells ± SEM compared to target cells without T-cells and antibody. **b** Flow cytometry of T_{TIS} -cells from co-cultures as in **a**. Bars represent mean percentage of $CD69^+$ cells ± SEM (gated on $CD3^+CD8^+$). **a**, **b** * $P < 0.05$ by two-sided, paired *t*-test. **c** Ratio (4-OHT divided by solvent) of DQ™ ovalbumin (DQ-OVA) antigen uptake and processing in control;*bcl2* lymphomas stably transduced with 4-hydroxytamoxifen (4-OHT)-inducible PU.1-ER^{T2} or C/EBPβ-ER^{T2} after treatment for 5 days (solvent [EtOH], ADR+EtOH, 4-OHT, or ADR+4-OHT). DQ-OVA signal measured by flow cytometry after a 2-hr incubation at 37°C. Mean fluorescence intensity (MFI) adjusted for background signal recorded in sample aliquots incubated at 4°C. **d** Viability of individual control;*bcl2* (*n* = 5) and *Suv39h1*⁻;*bcl2* (*n* = 3) lymphomas transduced with inducible

PU.1-ER^{T2} or C/EBPβ-ER^{T2}, treated as in **c** and then co-cultured with $T_{naïve}$ and T_{TIS} or T_{Suv} for 48-hr. Viability measured as 4-OHT/solvent ratio of viable cells in co-cultured lymphomas relative to the same cells in the absence of T-cells. * $P < 0.05$ by two-sided, one-sample *t*-test. **e** Flow cytometry of CD115 on individual control;*bcl2* ($n = 5$) and *Suv39h1*$^{-}$;*bcl2* ($n = 4$) lymphomas transduced with PU.1-ER^{T2} and treated as in **c.** **f** TIS lymphomas classification for T-cell priming in vivo (Fig. 8), based on CD115^{+} percentage. CD115^{high} defined as > 1% CD115^{+} cells (left). MFI ± SEM of CD86 on CD115^{low} and CD115^{high} TIS lymphomas (right). $n = 4$ each. Source data are provided in the Source Data file.

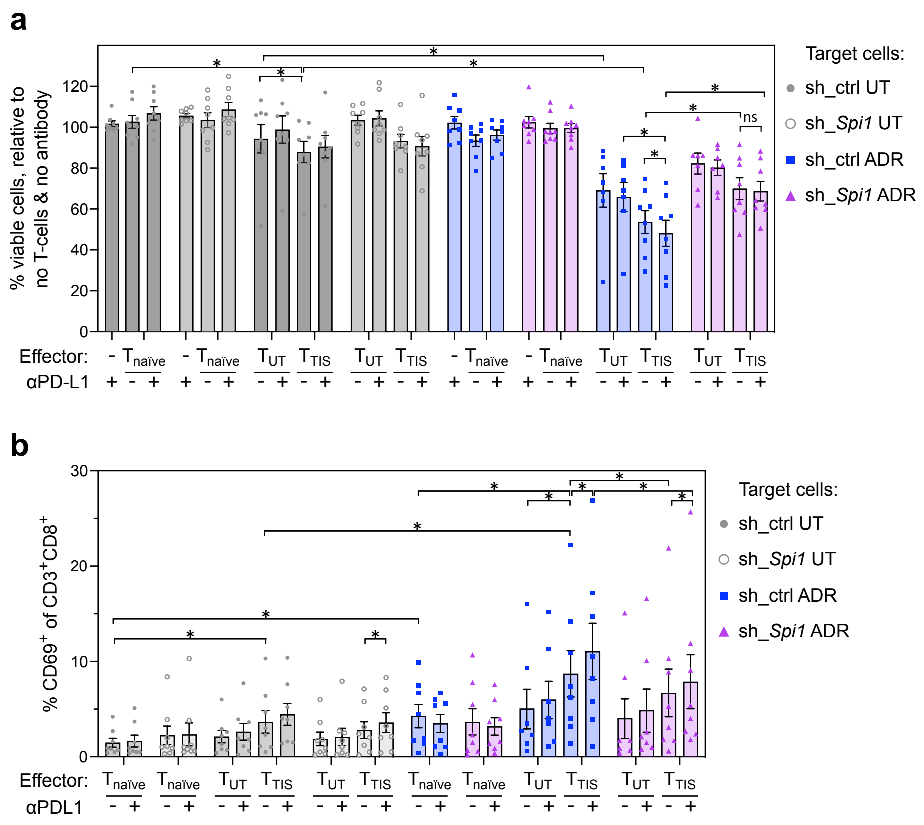

Supplementary Fig. 11: TIS-associated PU.1-evoked T-cell recognition is partially countered by immune checkpoint expression.

a, **b** Combined bar plots for data presented separately in Fig. 8b, c, e-h. All conditions for each individual lymphoma were run in parallel in the same experiment. **a** Viability of control;*bcl2* lymphomas, transduced with *Spi1* targeting or control shRNA (sh_ctrl) ($n = 8$ individual lymphomas each), following 6-day adriamycin (ADR)-exposure or left untreated (UT), then co-cultured for 72-hr with specified T-cell specimens, with or without PD-L1 blocking antibody (αPD-L1). Note that one T_{UT} specimen was not fully assessed due to residual GFP^+ lymphoma cells post-isolation that quickly overgrew co-cultures of ADR-treated lymphomas. **b** Flow cytometry of CD69 expression on $CD3^+CD8^+$ T-cells from co-cultures in **a**. Note that PD-L1-repressed TIS lymphomas (sh_ctrl ADR) co-cultured with TIS-primed T_{TIS} yielded the highest decrease in lymphoma viability and the highest $CD8^+$ T-cell activation. Source data are provided in the Source Data file.

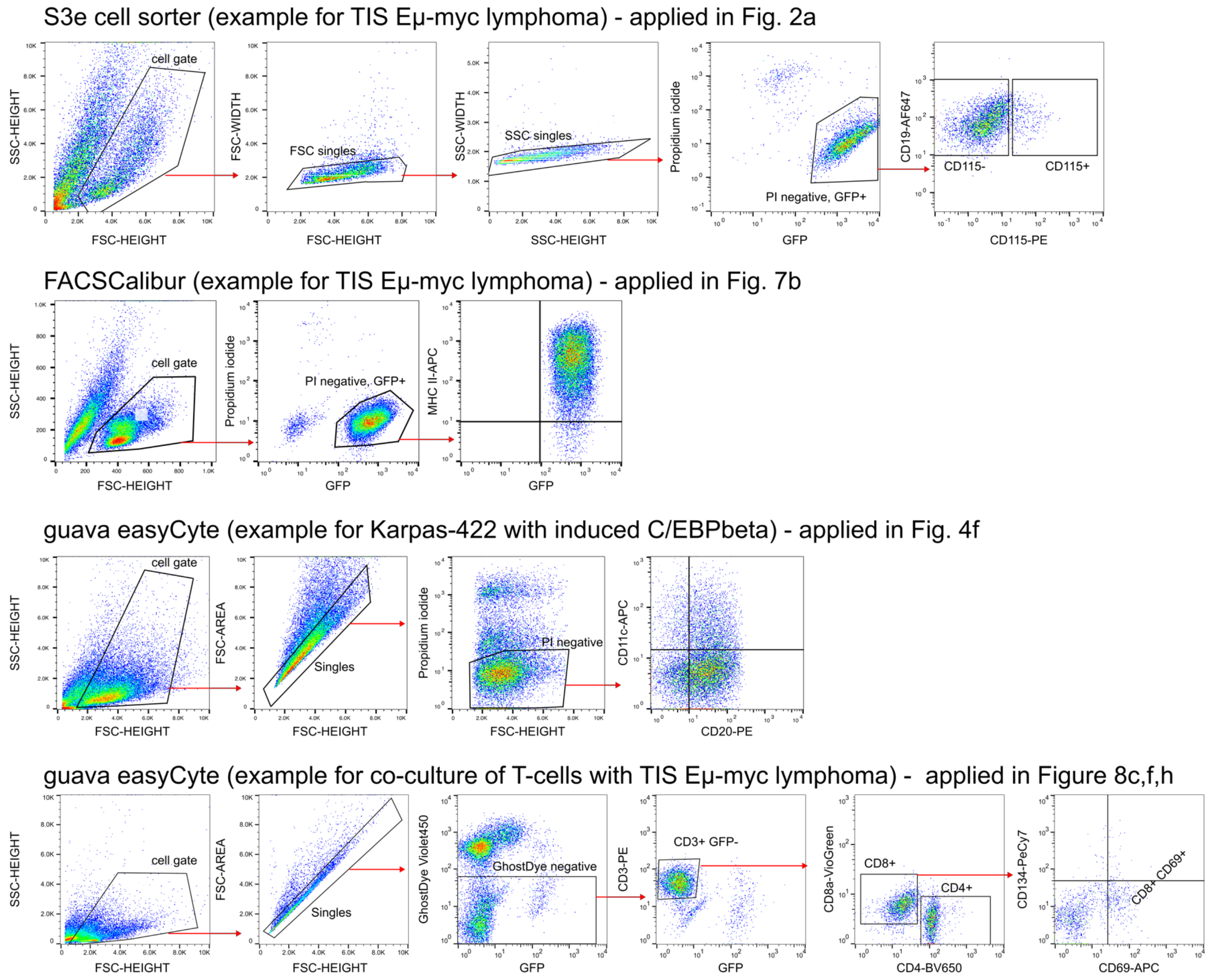

Supplementary Fig. 12: Flow cytometry gating strategies.

Gating strategies for data shown in figure panels as indicated.

Supplementary References

1 Martinez-Zamudio, R. I. et al. AP-1 imprints a reversible transcriptional programme of senescent cells. Nat Cell Biol 22, 842-855 (2020). https://doi.org/10.1038/s41556-020-0529-5

2 Huggins, C. J. et al. C/EBPγ suppresses senescence and inflammatory gene expression by heterodimerizing with C/EBPβ. Mol Cell Biol 33, 3242-3258 (2013). https://doi.org/10.1128/mcb.01674-12

3 Minderjahn, J. et al. Mechanisms governing the pioneering and redistribution capabilities of the non-classical pioneer PU.1. Nat Commun 11, 402 (2020). https://doi.org/10.1038/s41467-019-13960-2

4 Choi, J. et al. Haemopedia RNA-seq: a database of gene expression during haematopoiesis in mice and humans. Nucleic Acids Res 47, D780-d785 (2019). https://doi.org/10.1093/nar/gky1020

5 Schleich, K. et al. H3K9me3-mediated epigenetic regulation of senescence in mice predicts outcome of lymphoma patients. Nat Commun 11, 3651 (2020). https://doi.org/10.1038/s41467-020-17467-z

6 Wang, L. et al. High-Throughput Functional Genetic and Compound Screens Identify Targets for Senescence Induction in Cancer. *Cell Rep* 21, 773-783 (2017). https://doi.org/10.1016/j.celrep.2017.09.085

7 Jackson, J. G. et al. p53-mediated senescence impairs the apoptotic response to chemotherapy and clinical outcome in breast cancer. *Cancer Cell* 21, 793-806 (2012). https://doi.org/10.1016/j.ccr.2012.04.027

8 Pawlikowski, J. S. et al. Wnt signaling potentiates nevogenesis. *Proc Natl Acad Sci U S A* 110, 16009-16014 (2013). https://doi.org/10.1073/pnas.1303491110

9 Redmer, T. et al. JUN mediates the senescence associated secretory phenotype and immune cell recruitment to prevent prostate cancer progression. *Mol Cancer* 23, 114 (2024). https://doi.org/10.1186/s12943-024-02022-x

10 Lee, S. et al. Virus-induced senescence is driver and therapeutic target in COVID-19. Nature (2021). https://doi.org/10.1038/s41586-021-03995-1

11 Arango Duque, G. & Descoteaux, A. Macrophage cytokines: involvement in immunity and infectious diseases. Front Immunol 5, 491 (2014). https://doi.org/10.3389/fimmu.2014.00491

12 de Gruijter, N. M., Jebson, B. & Rosser, E. C. Cytokine production by human B cells: role in health and autoimmune disease. Clin Exp Immunol 210, 253-262 (2022). https://doi.org/10.1093/cei/uxac090

13 Chen, W. et al. Single-Cell Transcriptome Analysis Reveals Six Subpopulations Reflecting Distinct Cellular Fates in Senescent Mouse Embryonic Fibroblasts. Front Genet 11, 867 (2020). https://doi.org/10.3389/fgene.2020.00867

14 Reddy, A. et al. Genetic and Functional Drivers of Diffuse Large B Cell Lymphoma. Cell 171, 481-494 e415 (2017). https://doi.org/10.1016/j.cell.2017.09.027

15 Dreval, K. et al. Revisiting Reddy: A DLBCL Do-over. bioRxiv, 2023.2011.2021.567983 (2023). https://doi.org/10.1101/2023.11.21.567983
16 Lenz, G. et al. Stromal Gene Signatures in Large-B-Cell Lymphomas. N Engl J Med 359, 2313-2323 (2008).
17 Wright, G. W. et al. A Probabilistic Classification Tool for Genetic Subtypes of Diffuse Large B Cell Lymphoma with Therapeutic Implications. Cancer Cell 37, 551-568 e514 (2020). https://doi.org/10.1016/j.ccell.2020.03.015
18 Schmitz, R. et al. Genetics and Pathogenesis of Diffuse Large B-Cell Lymphoma. N Engl J Med 378, 1396-1407 (2018). https://doi.org/10.1056/NEJMoa1801445
19 Steen, C. B. et al. The landscape of tumor cell states and ecosystems in diffuse large B cell lymphoma. Cancer Cell (2021). https://doi.org/10.1016/j.ccell.2021.08.011
20 Jin, L. et al. MPYS, a novel membrane tetraspanner, is associated with major histocompatibility complex class II and mediates transduction of apoptotic signals. Mol Cell Biol 28, 5014-5026 (2008). https://doi.org/10.1128/mcb.00640-08
