# Supplementary material for: Senescence-associated lineage-aberrant plasticity evokes T-cell-mediated tumor control
Source: Nat Commun. 2025 Mar 31;16:3079. doi: 10.1038/s41467-025-57429-x (PMC11955568; doi:10.1038/s41467-025-57429-x)
Supplement: Supplementary file 2 — Description of Additional Supplementary Information [file 41467_2025_57429_MOESM2_ESM.pdf]

Description of Additional Supplementary Files

Supplementary Data 1:	Induced network modules analysis
Supplementary Data 2:	Immune deconvolution analyses
Supplementary Data 3:	sh_TF differential expression analyses
Supplementary Data 4:	DLBCL cell line ADR treatment and FACS data
Supplementary Data 5:	PU.1 targets in lymphoma TIS
Supplementary Data 6:	Antibodies and Taqman assays
Supplementary Data 7:	Central gene sets for gene set enrichment analyses
Source Data:	Statistical source data for all appropriate figures and uncropped Western and Simple Western blot images
